# Midlife Urinary Phthalate Metabolite Concentrations and Prior Uterine Fibroid Diagnosis

**DOI:** 10.3390/ijerph19052741

**Published:** 2022-02-26

**Authors:** Diana C. Pacyga, Brad A. Ryva, Romana A. Nowak, Serdar E. Bulun, Ping Yin, Zhong Li, Jodi A. Flaws, Rita S. Strakovsky

**Affiliations:** 1 Department of Food Science and Human Nutrition, Institute for Integrative Toxicology, Michigan State University, East Lansing, MI 48824, USA; pacygadi@msu.edu; 2 Department of Pharmacology and Toxicology, Institute for Integrative Toxicology, College of Osteopathic Medicine, Michigan State University, East Lansing, MI 48824, USA; ryvabrad@msu.edu; 3 Department of Animal Sciences, University of Illinois, Urbana-Champaign, IL 61801, USA; ranowak@illinois.edu; 4 Division of Reproductive Science in Medicine, Department of Obstetrics and Gynecology, Prentice Women’s Hospital, Northwestern University Feinberg School of Medicine, Chicago, IL 60611, USA; serdar.bulun@nm.org; 5 Division of Reproductive Science in Medicine, Department of Obstetrics and Gynecology, Northwestern University Feinberg School of Medicine, Chicago, IL 60611, USA; p-yin@northwestern.edu; 6 Roy J. Carver Biotechnology Center, University of Illinois, Urbana-Champaign, IL 61801, USA; lucasli@illinois.edu; 7 Department of Comparative Biosciences, University of Illinois, Urbana-Champaign, IL 61801, USA; jflaws@illinois.edu

**Keywords:** phthalates, endocrine disruptors, fibroids, leiomyoma, midlife, women

## Abstract

Fibroid etiology is poorly understood but is likely hormonally mediated. Therefore, we evaluated associations between midlife phthalates (hormone-altering chemicals) and prior fibroid diagnosis, and considered differences by weight gain status. Women (ages: 45–54; *n* = 754) self-reported past fibroid diagnosis. We pooled 1–4 urines collected after fibroid diagnosis over the consecutive weeks to analyze nine phthalate metabolites and calculate relevant molar sums (e.g., di(2-ethylhexyl) phthalate, ΣDEHP; anti-androgenic phthalates, ΣAA; all metabolites, ΣPhthalates). Using Poisson regression, we evaluated associations between phthalate biomarkers and the risk of having fibroid diagnosis. We explored if associations differed by weight gain from age 18 to 45–54 or in women diagnosed with fibroids within 5 years of phthalate assessment. Our major finding was that women had a 13% (RR: 1.13; 95%CI: 1.02, 1.26) and 16% (RR: 1.16; 95% CI: 1.03, 1.31) greater risk of prior fibroid diagnosis for each two-fold increase in ΣDEHP or ΣAA, respectively. These associations were strongest in women who became overweight/obese from age 18 to 45–54 and in those diagnosed <5 years before phthalate assessment. Based on these results, prospective studies should corroborate our findings related to associations between phthalates and fibroids, and may consider evaluating the role that weight gain may play in these associations.

## 1. Introduction

By midlife, most women will have uterine leiomyomata, commonly known as fibroids, which are non-cancerous tumors of uterine smooth muscle cells associated with adverse health outcomes, including abnormal uterine bleeding and miscarriage [1,2,3]. Fibroids almost exclusively occur in reproductive-aged, pre- and perimenopausal women, with incidence increasing with age until women are post-menopausal [2,4,5]. However, the exact prevalence of fibroids is difficult to determine because of the spectrum of clinical presentation. Many women with fibroids have a benign presentation, where fibroids are incidentally detected during imaging, whereas up to half of women with fibroids experience symptoms that are serious enough to impact quality of life, including excessive menstrual bleeding and pelvic pain [6,7]. With very severe symptoms, fibroids are the number one reason for hysterectomy in the United States [6]. Given the detrimental impacts of fibroids on women’s quality of life, substantially more data are needed to identify modifiable risk factors that contribute to the development of fibroids.

Pre- and perimenopausal women are widely exposed to endocrine-disrupting chemicals, including phthalates, which are found in many consumer products. For example, di(2-ethylhexyl) phthalate (DEHP) is a plasticizer used during food processing and in food contact materials, whereas diethyl phthalate (DEP) is used as a fragrance stabilizer in personal care products and cosmetics [8]. Given that phthalates are metabolized and excreted within 24–48 h of exposure, phthalate exposure is best approximated by measuring urinary concentrations of phthalate metabolites [9]. Although most individuals in the U.S. general population are exposed to phthalates [10], women have higher measured urinary levels of phthalate metabolites than men, likely due to the use of personal care products and cosmetics [11]. In women, studies have demonstrated that phthalates are associated with greater risk of hormone-mediated health outcomes, such as endometriosis [12], hot flashes [13], metabolic syndrome [14], breast cancer [15], and uterine fibroids [16]. In cell and experimental animal models, phthalates can alter circulating sex-steroid hormone concentrations by binding to hormone receptors, including peroxisome proliferator-activated receptor alpha receptors and estrogen receptors (alpha and beta) [17,18,19,20,21]. Similarly, in pregnant populations, as well as non-pregnant midlife women (ages 40 to 60), urinary phthalate biomarker concentrations were associated with altered serum and urinary sex-steroid hormone concentrations [22,23]. In addition to hormonal disruption, DEHP and its metabolites may disrupt other critical cellular processes, including cell viability and growth pathways, which may be responsible for the development of adverse health conditions in women, including uterine fibroids [24,25,26]. For example, an in vitro study reported that cells isolated from human uterine fibroids and treated with DEHP had higher viability, lower apoptosis, and increased expression of hypoxia-inducible factor-1α and cyclooxygenase-2 [26]. Additionally, a cross-sectional study of pre-menopausal women found that urinary concentrations of one DEHP metabolite, mono-(2-ethyl-5-hydroxyhexyl) phthalate (MEHHP), were associated with altered micro-RNA levels (miR-10a-5p and miR-577), which are important for cell viability, survival, and apoptosis in fibroid tumors, suggesting that phthalates may play a role in fibroid pathogenesis by interacting with regulators of epigenetic machinery [24]. Given this experimental evidence, additional studies in human populations are needed to determine whether these experimental findings recapitulate the real-life experience of women.

Many observational studies have investigated associations between phthalate metabolite biomarker concentrations and uterine fibroids [16,27,28,29]. A 2017 meta-analysis found no significant pooled associations between total urinary phthalate metabolite concentrations and uterine fibroids, whereas DEHP metabolites were associated with higher odds of fibroids [16]. Additional recent studies have confirmed positive associations between urinary DEHP metabolites, especially mono-2-ethylhexyl phthalate (MEHP), and fibroids [27,28]. A major limitation of these studies is that urine samples for phthalate biomarker assessment were collected after fibroid diagnosis, which makes it difficult to establish temporality and causality. A recent prospective study of 23–35-year-old premenopausal black women in Detroit that evaluated the associations between phthalates and ultrasound-detected fibroids (*n* = 301 women who did and 453 who did not develop fibroids) found a weak-to-moderate association between MEHP and higher risk of fibroids [29], supporting results from previous studies.

Similar to most previous studies, our current study did not prospectively evaluate associations between phthalate biomarker concentrations and fibroids. However, our goal was to contribute additional findings on the overall associations between phthalate biomarkers and fibroids in a large, diverse cohort of pre- and peri-menopausal midlife women, and also propose potential effect modification by adulthood weight gain, which (to our knowledge) has not been previously considered. Our first objective was to evaluate the overall associations between urinary phthalate biomarker concentrations and prior fibroid diagnosis. As weight change, specifically weight gain [30], is a risk factor for fibroid development, our second objective was to evaluate if associations between phthalate biomarkers and prior fibroid diagnosis differed in women who became overweight/obese from age 18 to midlife compared to women whose body mass index (BMI) remained stable (remained under-/normal weight or overweight/obese). To improve the window of our exposure measure relative to the outcome, our sensitivity analyses also considered whether associations between phthalate biomarkers and prior fibroid diagnosis differed based on the timing of diagnosis relative to phthalate assessment.

## 2. Materials and Methods

The current study was a secondary analysis of baseline data collected as part of the Midlife Women’s Health Study (MWHS). The MWHS is a prospective cohort that recruited midlife women living in and around Baltimore, Maryland, between 2006 and 2015, with the primary goal of assessing the risk factors of hot flashes. The study protocol has been described elsewhere [31]. In brief, women were included in the study if they were between 45 and 54 years old and were pre- or peri-menopausal. Women were excluded from the study if they had a history of hysterectomy or oophorectomy, were currently pregnant, were taking hormone therapy or herbal/other agents for menopause treatment, were taking oral contraceptives, were undergoing cancer treatment, or were postmenopausal. Menopause status was defined using the Stages of Reproductive Aging Workshop + 10 (STRAW+10) criteria, as follows [32]: premenopausal women were those who experienced their last menstrual period within the past three months and reported ≥11 periods within the past year; perimenopausal women were those who experienced their last menstrual period within the past year, but not within the past three months, or experienced their last menstrual periods within the past three months and experienced ≤10 periods within the past year; and postmenopausal women were those who had not experienced a menstrual period within the past year. Overall, 754 pre- and peri-menopausal women with complete information about baseline midlife urinary phthalate metabolite concentrations and self-reported past uterine fibroid diagnosis were available for the study.

At baseline, women reported their race/ethnicity, annual household income, alcohol intake, weight at age 18, oral contraceptive use, age at menarche, fertility consultation, and parity via a self-administered questionnaire. Women reported their race/ethnicity by selecting one of the following options: Caucasian/White, African American/Black, Hispanic, Asian, or other. To determine women’s most recent alcohol consumption status, women answered “yes” or “no” to the question, “In the last 12 months have you had at least 12 drinks of any kind of alcoholic beverage?”. Women reported “yes” or “no” if they ever used oral contraceptive pills. If they marked yes, they also reported the duration of use. To evaluate if women had reproductive problems, they answered “yes” or “no” to the question “Did you ever seek medical consultation because of difficulty in getting pregnant (infertility)?”. At the first baseline clinic visit, trained staff measured women’s height (inches) and weight (pounds), which we used to calculate midlife BMI (kg/m^2^). We additionally used measured midlife height and self-reported weight at age 18 to calculate BMI at age 18 (kg/m^2^).

Unfortunately, this study was not designed to assess fibroid incidence. Instead, the baseline questionnaire collected information about prior fibroid diagnosis. Women answered “yes” or “no” to the question “Have you ever been told by a doctor that you have uterine fibroids? If women reported “yes”, they indicated their age at diagnosis. We evaluated prior fibroid diagnosis as a binary variable comparing women who had a diagnosis to those without a diagnosis. We evaluated the timing of fibroid diagnosis as a three-level variable using the following categories: women who were diagnosed with fibroids within five years of the baseline visit, those diagnosed with fibroids five years or more before the baseline visit, and those never diagnosed with fibroids.

We were unable to ascertain women’s phthalate exposure at the time of their fibroid diagnosis. Instead, at the baseline clinic visit, women provided a urine sample and then provided up to three more urine samples over consecutive weeks. Staff physically pooled urine samples for each participant for analysis of phthalate metabolite biomarkers, which were used to approximate midlife exposure to these chemicals. Given the short half-lives of phthalates in the body and high within-person variability, pooling has been shown to be an effective approach for reducing measurement error in phthalate biomarkers [33]. MWHS staff sent one pooled urine sample per participant to the University of Illinois Urbana-Champaign Roy K. Carver Biotechnology Metabolomics Center for analysis. The metabolomics laboratory used isotope dilution high-performance liquid chromatography negative-ion electrospray ionization-tandem mass spectrometry (HPLC–MS/MS) and methods adapted from the Centers for Disease Control and Prevention [34] to analyze urine samples for concentrations (ng/mL) of the following nine phthalate metabolites: MEHP, MEHHP, mono-(2-ethyl-5-carboxypentyl) phthalate (MECPP), mono-(2-ethyl- 5-oxohexyl) phthalate (MEOHP), mono-(3-carboxypropyl) phthalate (MCPP), monobenzyl phthalate (MBzP), monoethyl phthalate (MEP), monobutyl phthalate (MBP), and monoisobutyl phthalate (MiBP).

We imputed phthalate metabolite concentrations below the limit of detection (LOD) using LOD/√2. All phthalate metabolite concentrations were specific gravity-adjusted to account for urine dilution using the following equation: *P*_c_ = *P*[(1.018 − 1)/(*SG* − 1)], where *P*_c_ is the specific gravity-adjusted metabolite concentration, *P* is the measured metabolite concentration (ng/mL), 1.018 is the median specific gravity of the MWHS population included in this analysis, and *SG* is the specific gravity of each woman’s pooled urine sample [35]. We molar-converted and summed (mmol/mL) four DEHP metabolites (MEHP, MEHHP, MEOHP, and MECPP) to approximate exposure to DEHP (referred to as ΣDEHP). The remaining metabolites (MCPP, MBzP, MEP, MBP, and MiBP) were evaluated using non-molar converted concentrations (ng/mL). We created additional molar sums based on exposure sources, where metabolites MEHP, MEHHP, MEOHP, MECPP, MCPP, and MBzP were molar-summed to approximate exposure to plasticizer phthalates (ΣPlastics) and metabolites MEP, MBP, and MiBP were molar-summed to approximate exposure to personal-care-product phthalates (ΣPCP). Some previous experimental studies in male pups suggest that metabolites MEHP, MEHHP, MEOHP, MECPP, MBzP, MBP, and MiBP have anti-androgenic activity in the body [36,37,38]. Therefore, we molar summed these urinary biomarkers to approximate exposure to phthalates with anti-androgenic activity (ΣAA). Interestingly, in our previous study, we observed positive associations between ΣAA and estradiol [23], suggesting that classifying phthalate biomarkers based on their effects on male fetal rat testes may not be appropriate for evaluating reproductive endpoints in women. However, we evaluated associations between ΣAA and fibroids to corroborate findings from a previous study [27]. Lastly, we molar-summed all nine phthalate metabolites to approximate total midlife phthalate exposure (ΣPhthalates).

We used the chi-squared test to evaluate differences in sociodemographic, lifestyle, and health characteristics between women with and without prior fibroid diagnosis. We then used Poisson regression models with robust variance estimator to evaluate associations between phthalate biomarker concentrations (as individual metabolites or molar sums) and prior fibroid diagnosis [39]. Due to skewed distributions, phthalate biomarker concentrations were natural log-transformed. To address our first and second objectives, we specified Poisson regression models to evaluate associations between phthalate biomarker concentrations and the risk of having a prior fibroid diagnosis compared to not having a prior fibroid diagnosis. To evaluate differences in associations between phthalate biomarker concentrations and prior fibroid diagnosis by changes in BMI from age 18 to 45–54 (second objective), we first classified both midlife BMI and BMI at age 18 using the following clinical categories [40]: under-/normal weight (<25 kg/m^2^) and overweight/obese (≥25 kg/m^2^). Then, we categorized changes in BMI as follows: women who remained overweight/obese through age 45–54 (overweight/obese at ages 18 and 45–54), women who became overweight/obese by age 45–54 (under-/normal weight at age 18 but overweight/obese at age 45–54), women who remained under-/normal weight through age 45–54 (under-/normal at ages 18 and 45–54), and those who became under-/normal weight by age 45–54 (overweight/obese at age 18 but under-/normal weight at age 45–54) [41]. In these models, we included a multiplicative interaction between phthalates and change in BMI to evaluate differences in associations in women who remained overweight/obese through age 45–54, who became overweight/obese by age 45–54, and who remained under-/normal weight. We excluded women who became under-/normal weight because this category only included five women. We reported results regardless of the significance of the interaction *p*-value.

We acknowledge that our study was not designed to prospectively evaluate the associations between urinary phthalate biomarker concentrations and fibroids diagnosis, and that midlife urinary phthalate biomarker concentrations likely do not represent concentrations at the time of prior fibroid diagnosis. However, in addition to our main analyses, we also conducted a sensitivity analysis to potentially provide a more relevant approximation of phthalate exposure in relation to prior fibroid diagnosis. Specifically, we wanted to assess whether our primary associations differed based on when women were diagnosed with fibroids (timing) relative to when they provided their midlife urine samples for phthalate metabolite quantification. We used multinomial logistic regression models to evaluate the associations between urinary phthalate biomarker concentrations and the probability of being diagnosed with fibroids within five years of or more than five years before midlife urine collection compared to never being diagnosed with fibroids.

For our first objective, we assessed both unadjusted and adjusted models. In adjusted models, we a priori selected covariates associated with both our exposure and outcome. We evaluated correlations between all selected covariates to test for potential multicollinearity issues, but none were strongly correlated with each other (*r* < 0.4; data not shown). Therefore, final adjusted models included the following covariates: race/ethnicity, annual household income, alcohol intake, fertility consultation, midlife BMI, oral contraceptive use, age at menarche, and parity. These covariates are proxies of important latent constructs that we were unable to directly assess at the time of fibroid diagnosis, such as socioeconomic status (race/ethnicity, income), racism (race/ethnicity), lifestyle (midlife BMI, alcohol use), health (midlife BMI, alcohol use, age at menarche, fertility consultation), and reproductive history (oral contraceptive use, parity, age at menarche, fertility consultation). These covariates were also accounted for in sensitivity analyses. For our second objective, we included the previously listed covariates, except for midlife BMI, due to multicollinearity issues with changes in BMI. The operationalization of covariates and reference groups are presented in Table 1.

We conducted all analyses in SAS 9.4 (SAS Institute Inc, Cary, NC, USA). We used PROC GENMOD for Poisson regression analyses with a robust variance estimator (main analyses) and specified an unstructured correlation matrix for the model’s residuals. We back-transformed the resulting risk ratios (RR) and 95% confidence intervals (CI) using the equation [exp(ln(RR)×ln(2.00)] to interpret the results as risk of prior fibroid diagnosis with every two-fold increase in phthalate biomarker concentration. We used PROC LOGISTIC for multinomial logistic regression models (sensitivity analysis), and back-transformed the resulting odds ratios (OR) and 95% CIs using the equation [exp(ln(OR) × ln(2.00)] to interpret the results as odds of being diagnosed with fibroids within five years, or more than five years before, midlife urine collection with every two-fold increase in phthalate biomarker concentration. We considered associations as being meaningful at *p* ≤ 0.10 and analyses were not adjusted for multiple comparisons [42].

## 3. Results

### 3.1. Demographic and Lifestyle Characteristics of the MWHS Population

The characteristics of the overall MWHS population have been described elsewhere [13,23]. The prevalence of prior fibroid diagnosis in MWHS was approximately 27% (Table 1), and median age at diagnosis was 40 years (range: 16–52; data not shown). Women with and without prior fibroid diagnosis differed significantly with regard to race/ethnicity, annual household income, alcohol intake, midlife BMI, age at menarche, oral contraceptive use, fertility consultation, and parity (*p* < 0.05; Table 1). Specifically, compared to women with no prior fibroid diagnosis, women with prior diagnosis were more likely to be black/other, have lower annual household incomes, have obesity during midlife, start menarche earlier, have no or one live birth, use oral contraceptives for >10 years, consume ≤12 alcoholic drinks in the year before the first study visit, and not to have received fertility treatment when trying to become pregnant.

### 3.2. Urinary Phthalate Metabolite Biomarker Concentrations

**Figure 1:**
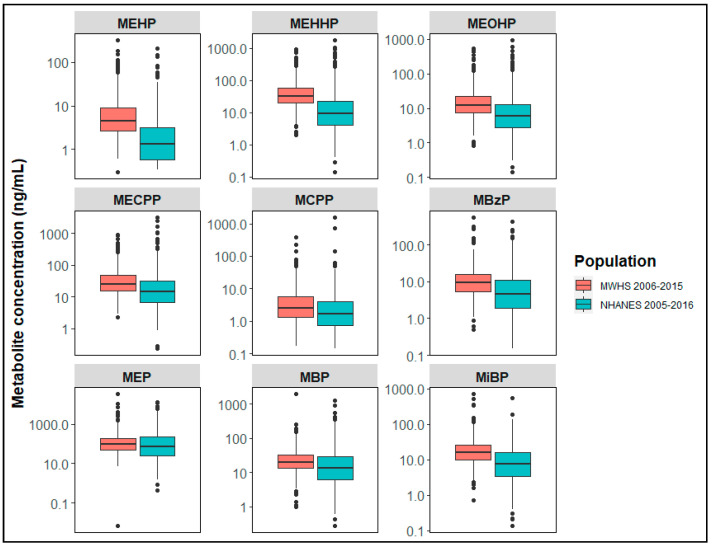
Midlife urinary phthalate metabolite concentrations. Box plots display urinary phthalate metabolite concentrations (ng/mL) of women in MWHS (2006–2015, *n* = 754) and women ages 45–54 from 6 NHANES survey cycles (2005–2016, *n* = 902). Concentrations were not adjusted for urine dilution. Box plots include the median (center line in box), the 25th percentile (lower line of box), and the 75th percentile (upper line in box). Numeric values for phthalate metabolite concentrations have been published elsewhere [13,23]. MEHP (mono-2-ethylhexyl phthalate); MEHHP (mono-(2-ethyl-5-hydroxyhexyl) phthalate); mono-(2-ethyl- 5-oxohexyl) phthalate (MEOHP); mono-(2-ethyl-5-carboxypentyl) phthalate (MECPP); mono-(3-carboxypropyl) phthalate (MCPP); monobenzyl phthalate (MBzP); monoethyl phthalate (MEP); monobutyl phthalate (MBP); and monoisobutyl phthalate (MiBP); MWHS, Midlife Women’s Health Study; NHANES, National Health and Nutrition Examination Survey.

More than 99% of women had urinary concentrations of all measured phthalate metabolites above the LOD (data not shown). MWHS women had somewhat higher median phthalate metabolite concentrations compared to midlife women from the National Health and Nutrition Examination Survey (NHANES) for the years 2005–2016, but with overlapping 25th and 75th percentiles [13,23] (Figure 1).

### 3.3. Associations of Phthalate Biomarker Concentrations with Prior Fibroid Diagnosis

Overall, higher concentrations of some phthalate biomarkers were associated with higher risk of prior fibroid diagnosis (Table 2). In unadjusted models, women had 10–19% higher risk of prior fibroid diagnosis for every two-fold increase in ΣDEHP (RR: 1.10; 95% CI: 1.00, 1.22), MEP (RR: 1.11; 95% CI: 1.02, 1.20), MiBP (RR: 1.18; 95% CI: 1.06, 1.31), ΣPCP (RR: 1.16; 95% CI: 1.08, 1.25), ΣPhthalates (RR: 1.19; 95% CI: 1.10, 1.29), and ΣAA (RR: 1.15; 95% CI: 1.02, 1.29). Interestingly, in unadjusted models, we also observed a marginal inverse association of MBzP with prior fibroid diagnosis (RR: 0.92; 95% CI: 0.84, 1.02). After accounting for important confounders, only associations of ΣDEHP, ΣPhthalates, and ΣAA with fibroids remained. Women had a 9–16% higher risk of prior fibroid diagnosis, respectively, for every two-fold increase in ΣDEHP (RR: 1.13; 95% CI: 1.02, 1.26), ΣPhthalates (RR: 1.09; 95% CI: 1.00, 1.19), or ΣAA (RR: 1.16; 95% CI: 1.03, 1.31). In adjusted models, we also observed a marginal association with ΣPlastics, where women had a 12% (RR: 1.12; 95% CI: 1.00, 1.25) higher risk of prior fibroid diagnosis for every two-fold increase in ΣPlastics.

### 3.4. Differences in Associations by Change in BMI from 18 Years of Age to Midlife

**Figure 2:**
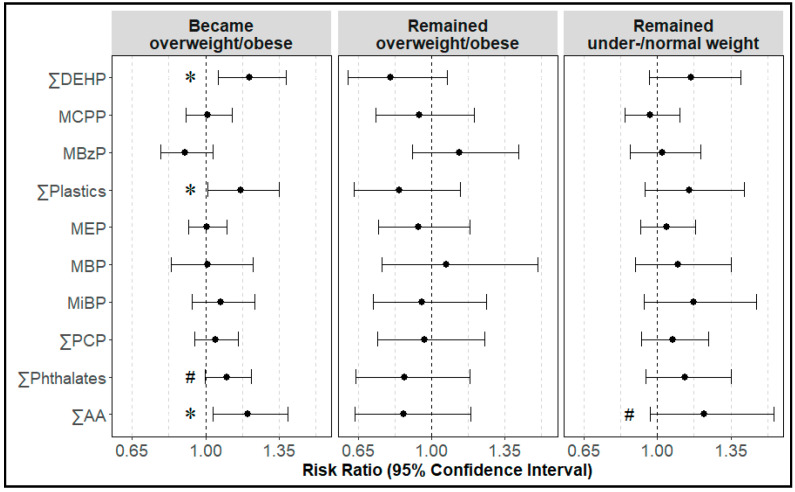
Differences in associations between phthalate biomarker concentrations and uterine fibroids by change in BMI from age 18 to 45–54. Forest plots display risk ratios (filled circle) and 95% confidence intervals (horizonal lines) for the risk of prior fibroid diagnosis for every 2-fold increase in phthalate biomarker concentrations among women who remained overweight/obese (*n* = 223 no prior fibroids diagnosis, *n* = 66 yes prior fibroids diagnosis), became overweight/obese (*n* = 135 no prior fibroids diagnosis, *n* = 47 yes prior fibroids diagnosis), and remained under-/normal weight (n=161 no prior fibroids diagnosis, *n* = 80 yes prior fibroids diagnosis) from age 18 to 45–54. Models account for race/ethnicity, income, age at menarche, oral contraceptive use, parity, fertility consultation, change in BMI from age 18 to 45–54, and a multiplicative interaction between phthalate biomarker and change in BMI. Confidence intervals that do not cross the null (dashed vertical line) are significant at ^#^ *p* ≤ 0.10 or * *p* < 0.05. ∑DEHP = MEHP/278 + MEHHP/294 + MEOHP/292 + MECPP/308; ∑Plastics = MEHP/278 + MEHHP/294 + MEOHP/292 + MECPP/308 + MCPP/252 + MBzP/256; ∑PCP = MEP/194 + MBP/222 + MiBP/222; ∑Phthalates = MEHP/278 + MEHHP/294 + MEOHP/292 + MECPP/308 + MCPP/252 + MBzP/256 + MEP/194 + MBP/222 + MiBP/222; ∑AA = MEHP/222 + MEHHP/294 + MEOHP/292 + MECPP/308 + MBzP/256 + MBP/222 + MiBP/222.

Associations of phthalate biomarker concentrations with prior fibroid diagnosis were strongest in women who became overweight or obese (Figure 2). Specifically, women who became overweight or obese had a 17–21% higher risk of having a prior fibroid diagnosis for every two-fold increase in ΣDEHP (RR: 1.21; 95% CI: 1.06, 1.38), ΣPlastics (RR: 1.17; 95% CI: 1.01, 1.35), and ΣAA (RR: 1.20; 95% CI: 1.04, 1.39). Additionally, we observed a marginal association between ΣPhthalates and fibroids, where women who became overweight or obese had a 10% (RR: 1.10; 95% CI: 1.00, 1.22) higher risk of having a prior fibroid diagnosis for every two-fold increase in ΣPhthalates. Lastly, we observed a marginal association between ΣAA and fibroids, where women who remained under-/normal weight had a 22% (RR: 1.22; 95% CI: 0.96, 1.55) higher risk of having a prior fibroid diagnosis for every two-fold increase in ΣAA.

### 3.5. Associations of Phthalate Biomarker Concentrations in Women with More Recent Diagnosis

Among women who had a prior fibroids diagnosis, median time of diagnosis relative to midlife urine collection for phthalate biomarker assessment was 8 years (range: 0–33 years). In sensitivity analyses, the associations between some phthalate biomarkers and fibroid diagnosis differed according the recency of diagnosis relative to urine collection for phthalate biomarker assessment (Table 3). Overall associations between ΣDEHP, ΣPlastics, ΣPhthalates, ΣPCP, and ΣAA and prior fibroid diagnosis were more robust in women diagnosed within five years of midlife urine collection. Specifically, women diagnosed within five years of midlife urine collection generally had 19–35% higher odds of prior fibroid diagnosis for every two-fold increase in ΣDEHP (OR: 1.29; 95% CI: 1.03, 1.61), ΣPlastics (OR: 1.23; 95% CI: 0.96, 1.56), ΣPCP (OR: 1.30; 95% CI: 0.99, 1.43), ΣPhthalates (OR: 1.30; 95% CI: 1.05, 1.61), and ΣAA (OR: 1.35; 95% CI: 1.04, 1.76).

## 4. Discussion

Overall, we observed that ΣDEHP was associated with a higher risk of prior fibroid diagnosis, which was the main contributor to the associations between ΣPlastics, ΣPhthalates, and ΣAA and prior fibroid diagnosis. These associations were strongest in women who became overweight/obese from ages 18 to 45–54. The associations between phthalate biomarker concentrations and prior fibroid diagnosis were also stronger in women diagnosed with fibroids within five years of midlife urine collection for phthalate biomarker assessment. Our overall results related to ΣDEHP with prior fibroid diagnosis corroborate those from previous studies. However, additional, large-scale prospective studies in diverse populations are needed.

Our findings that ΣDEHP and related phthalate molar sums are associated with higher odds of prior fibroid diagnosis are consistent with previous experimental and observational studies. Specifically, experimental studies observed that human fibroid cells treated with DEHP metabolites had disrupted cell viability, apoptotic, and growth pathways [24,25,26]. A 2017 meta-analysis pooled five observational studies conducted between 1999 and 2015 in populations from the U.S., Korea, China, and Taiwan, and observed that DEHP metabolites were associated with higher odds of fibroids [16]. However, this meta-analysis also reported that MBP and MiBP were also marginally significantly associated with higher odds of fibroids. These differences in findings may be related to the study population, since women in the meta-analysis were predominately non-Hispanic white or Asian, while most women in our population were non-Hispanic white or black. Additionally, our study population included women between the ages of 45 and 54, while the meta-analysis included a wider age range of women. Our findings are also consistent with a recent U.S. cross-sectional study of predominately black 26–54-year-old women (recruited 2014–2017) undergoing hysterectomy or myomectomy that reported positive associations between ΣDEHP and ΣAA and fibroid volume [27]. Similarly, a case-control study of 20–40-year-old Korean women (recruited 2015–2016) reported that the odds of fibroids was higher in women in quartiles 2, 3, and/or 4 (compared to quartile 1) of urinary concentrations of ƩDEHP and its metabolites [28]. These two more recent studies collected spot urine samples for phthalate biomarker assessment and ascertained fibroid status using imaging technology (i.e., magnetic resonance imaging (MRI), ultrasound) and/or pathology reports [27,28]. Despite the differences in study population, as well as phthalate biomarker and fibroid assessment, in these studies compared to ours, associations between DEHP and its metabolites and fibroids appear to be consistent.

The causal interpretability of results from most studies evaluating the associations of phthalate metabolite biomarkers with fibroids is limited because urine collection for phthalate biomarker assessment often occurred after women had already been diagnosed with fibroids. To our knowledge, only one study of Detroit-area 23–35-year-old black women (recruited 2010–2012) prospectively evaluated associations between phthalate biomarkers and incidence of fibroids [29]. Although we were also unable to prospectively evaluate these associations, we conducted sensitivity analyses to address this limitation. In sensitivity analyses assessing the timing of fibroid diagnosis relative to midlife urine collection for phthalate biomarker assessment, we found that associations of ΣDEHP and related phthalate molar sums with higher likelihood of prior fibroid diagnosis were stronger in women diagnosed within five years of urine collection. This five-year cutoff makes it more likely that women’s urinary phthalate biomarker concentrations at the time of fibroid diagnosis were similar to those during the study. However, even with these sensitivity analyses, we cannot rule out the potential of reverse causation. Women diagnosed with fibroids may have developed certain lifestyles or behaviors over the years leading up to their enrollment into the MWHS that may have influenced their midlife exposure to phthalates. Additionally, given the high temporal variability in urinary phthalate biomarker concentrations [43], we are likely not capturing the correct exposure window, which may result in the underestimation of associations between phthalate biomarkers and risk of fibroids. Therefore, additional prospective studies are needed to elucidate the directionality of associations between phthalate biomarkers and fibroid development, and to consider the sensitive window of exposure framework, to establish the temporal relationship between phthalate exposure and fibroid development. Nevertheless, the findings from this study should be interpreted with caution.

To our knowledge, our study is the first to show that associations between ΣDEHP and related phthalate molar sums and prior fibroid diagnosis were driven by women who became overweight or obese from age 18 to midlife. Obesity and especially weight gain are important risk factors for the development of fibroids. A recent meta-analysis that pooled 22 studies observed that higher body weight and adiposity (measured by BMI, waist/hip ratio, and waist circumference), as well as weight gain since age 18, were associated with higher odds of fibroids [44]. Given that both phthalates and gaining weight since age 18 are determinants of fibroid development, our findings suggest that women who undergo major changes in weight may be more susceptible to the impact of phthalates than those whose weights remain stable from age 18 until midlife. These results could be due to the metabolic disruptions that occur with weight change. For example, changes in weight/fat distribution and reproductive hormones (i.e., estrogens) are linked, with obesity influencing hormone concentrations [45,46], as well as reproductive hormones influencing changes in weight and fat deposition [47]. Given this relationship between body composition/adiposity and hormones, the interaction between endocrine disrupting chemicals (i.e., phthalates) and adipose tissue may contribute to the increased risk of phthalate-induced fibroids in women who gained weight. While most individuals experience gradual weight gain across the lifespan [48], because we did not have information about women’s weight at fibroid diagnosis, it is possible that some weight gain may have occurred after diagnosis. Additionally, women who gained weight may have engaged in certain behaviors or have lifestyles resulting in the use of products associated with increased exposure to DEHP, which may explain why associations between ΣDEHP and related phthalate molar sums and prior fibroid diagnosis were stronger in these women. For example, unhealthy dietary behaviors, such as consumption of processed and fast foods, are major determinants of phthalate exposure [8]. A study using data from NHANES found that individuals who consumed more fast foods had higher urinary ƩDEHP concentrations than non-consumers [49]. Future studies that consider diet are needed to corroborate our findings, and to address biologically plausible pathways connecting phthalate exposure and weight change with the development of fibroids.

This current study has important strengths, but also some limitations, in addition to the potential for reverse causation. First, we may be underpowered to detect certain associations of phthalate biomarkers with prior fibroid diagnosis, especially in sensitivity analyses and in analyses evaluating differences by changes in BMI from age 18 to midlife. However, we evaluated these cross-sectional associations in a large cohort of pre- and peri-menopausal midlife women with a relatively high prevalence of fibroids. Second, prior fibroid diagnosis was based on self-reports and women with a history of hysterectomy or oophorectomy were excluded, which could lead to the misclassification of fibroid status and underestimation of the number of women with fibroids [50]. However, our findings that race/ethnicity, BMI, and age at menarche are important predictors of self-reported fibroid diagnosis are consistent with the prior literature [51]. Additionally, our results related to the associations between ƩDEHP and prior fibroid diagnosis are also consistent with previous studies that determined fibroid status using imaging technology (i.e., MRI, ultrasound) and/or pathology reports [27,28]. Third, we used self-reported weight at age 18 to calculate changes in BMI from age 18 to midlife, which may also be subject to recall bias. However, at the population level, self-reported past body weight is reliable for predicting measured past body weight [52]. Fourth, we were unable to causally interpret our results given that urine for midlife phthalate biomarker assessment was collected after women’s fibroid diagnosis. However, we conducted sensitivity analyses and observed that associations between phthalate biomarkers and fibroids are stronger when the time between midlife urine collection and prior fibroid diagnosis is reduced. Additionally, phthalate metabolite concentrations were quantified from pools of up to four urine samples, which provides a more stable measure of our exposure and may better represent midlife urinary concentrations [33,53]. Lastly, as previously discussed, there may be unmeasured confounding factors (i.e., by diet), which were unaccounted for in our statistical models evaluating associations between phthalate biomarkers and prior fibroid diagnosis. However, we selected covariates a priori using the previous literature, and included covariates that are proxies for important latent constructs that we were unable to directly assess at the time of fibroid diagnosis. Overall, this secondary data analysis leverages data collected as part of the MWHS to contribute additional information pertaining to associations between phthalate biomarkers and prior fibroid diagnosis in midlife women.

## 5. Conclusions

In this population of mostly non-Hispanic white or black pre- and peri-menopausal midlife women, we observed that ΣDEHP and related phthalate molar sums were associated with higher risk of prior fibroid diagnosis, and these associations were stronger in women who became overweight or obese from age 18 to midlife. Our findings corroborate results from previous experimental and observational studies, and suggest that interventions targeting the lifestyle behaviors associated with phthalate exposure are needed. However, as with most previous studies, we were unable to prospectively evaluate these associations, which makes it challenging to causally interpret our results, and our findings should be interpreted with caution. Therefore, future longitudinal, prospective cohort studies are needed to corroborate these findings and further elucidate the independent and interactive contribution of phthalates and weight gain to the development of uterine fibroids.

## Figures and Tables

**Table 1:** Demographic and lifestyle characteristics of women with and without uterine fibroids.

	Fibroid Diagnosis		
Participant Characteristic	Yes (*n* = 207)	No (*n* = 547)	*p*-Value
Age at baseline			0.49
45–49 years	131 (63.3)	361 (66.0)	
50–54 years	76 (36.7)	186 (34.0)	
Race/ethnicity			<0.0001
Non-Hispanic white	88 (42.7)	407 (74.5)	
Black/other ^1^	118 (57.3)	139 (25.5)	
Employment			0.94
Unemployed	41 (19.9)	110 (20.1)	
Employed	165 (80.1)	436 (79.9)	
Educational attainment			0.25
Some college or less	79 (38.5)	186 (34.1)	
College graduate or higher	126 (61.5)	360 (65.9)	
Annual household income			0.01
<$20,000	14 (7.0)	35 (6.6)	
$20,000–39,999	41 (20.4)	78 (14.7)	
$40,000–99,999	78 (38.8)	169 (31.8)	
≥$100,000	68 (33.8)	249 (46.9)	
Marital status			0.21
Single	42 (20.5)	92 (16.8)	
Married/living with partner	124 (60.5)	368 (67.4)	
Widowed/divorced/separated	39 (19.0)	86 (15.8)	
Alcohol intake			0.001
No	91 (44.2)	169 (31.0)	
Yes	115 (55.8)	376 (69.0)	
Ever smoker			0.31
Yes	86 (41.7)	251 (45.9)	
No	120 (58.3)	296 (54.1)	
Menopause status			0.61
Premenopausal	137 (66.2)	351 (64.2)	
Perimenopausal	70 (33.8)	196 (35.8)	
Midlife BMI			0.02
<25 kg/m^2^	70 (33.8)	230 (42.0)	
25–29.9 kg/m^2^	52 (25.1)	149 (27.2)	
≥30.0 kg/m^2^	85 (41.1)	168 (30.7)	
Age at menarche			0.001
<12 years	119 (58.0)	231 (42.5)	
13–14 years	64 (31.2)	238 (43.8)	
≥15 years	22 (10.7)	74 (13.6)	
Oral contraceptive use			0.04
Never	24 (11.7)	86 (15.8)	
<1 year	35 (17.1)	70 (12.8)	
1–4 years	51 (24.9)	146 (26.8)	
5–10 years	40 (19.5)	139 (25.5)	
>10 years	55 (26.8)	104 (19.1)	
Fertility consultation			0.03
Yes	28 (13.7)	111 (20.4)	
No	177 (86.3)	433 (79.6)	
Parity			0.03
Never pregnant	20 (9.7)	66 (12.1)	
No live births	25 (12.1)	50 (9.2)	
1 live birth	49 (23.8)	86 (15.8)	
≥2 live births	112 (54.4)	344 (63.0)	
Change in BMI from age 18 to 45–54			0.29
Remained under-/normal weight	68 (33.7)	224 (41.2)	
Became overweight/obese	107 (53.0)	253 (46.5)	
Became under-/normal weight	1.0 (0.5)	4 (0.7)	
Remained overweight/obese	26 (12.9)	63 (11.6)	

^1^ Women of another race/ethnicity represent less than 4% of the analytic sample. *p*-value from chi-squared test. Missing information (*n*) for women with prior fibroid diagnosis: race/ethnicity, employment, alcohol intake, ever smoker, parity (*n* = 1); education, marital status, age at menarche, oral contraceptive use, fertility consultation (*n* = 2); change in BMI from age 18 to 45–54 (*n* = 5); income (*n* = 6). Missing information (*n*) for women without prior fibroid diagnosis: race/ethnicity, employment, education, marital status, parity (*n* = 1); alcohol intake, oral contraceptive use (*n* = 2); fertility consultation, change in BMI from age 18 to 45–54 (*n* = 3); age at menarche (*n* = 4); income (*n* = 16). BMI, body mass index.

**Table 2:** Associations between phthalate biomarker concentrations and diagnosis of uterine fibroids.

	Unadjusted (*n* = 754)	Adjusted (*n* = 712)
Phthalate Biomarker	RR (95% CI)	RR (95% CI)
∑DEHP ^1^	1.10 (1.00, 1.22) ^#^	1.13 (1.02, 1.26) *
MCPP	0.99 (0.92, 1.07)	0.99 (0.91, 1.07)
MBzP	0.92 (0.84, 1.02) ^#^	0.97 (0.88, 1.07)
∑Plastics ^2^	1.08 (0.97, 1.20)	1.12 (1.00, 1.25) ^#^
MEP	1.11 (1.02, 1.20) *	1.01 (0.94, 1.09)
MBP	1.06 (0.94, 1.20)	1.05 (0.94, 1.19)
MiBP	1.18 (1.06, 1.31) *	1.09 (0.98, 1.22)
∑PCP ^3^	1.16 (1.08, 1.25) *	1.05 (0.97, 1.14)
∑Phthalates ^4^	1.19 (1.10, 1.29) *	1.09 (1.00, 1.19) *
∑AA ^5^	1.15 (1.02, 1.29) *	1.16 (1.03, 1.31) *

Poisson regression models with robust variance estimator evaluating the risk of being diagnosed with fibroids (unadjusted model *n* = 207, adjusted model *n* = 193) compared to never being diagnosed with fibroids (unadjusted model *n* = 547, adjusted model *n* = 519) for every 2-fold increase in phthalate biomarker concentration. Adjusted models account for race/ethnicity, income, age at menarche, oral contraceptive use, parity, fertility consultation, and midlife BMI. ^1^ ∑DEHP = MEHP/278 + MEHHP/294 + MEOHP/292 + MECPP/308; ^2^ ∑Plastics = MEHP/278 + MEHHP/294 + MEOHP/292 + MECPP/308 + MCPP/252 + MBzP/256; ^3^ ∑PCP = MEP/194 + MBP/222 + MiBP/222; ^4^ ∑Phthalates = MEHP/278 + MEHHP/294 + MEOHP/292 + MECPP/308 + MCPP/252 + MBzP/256 + MEP/194 + MBP/222 + MiBP/222; ^5^ ∑AA = MEHP/222 + MEHHP/294 + MEOHP/292 + MECPP/308 + MBzP/256 + MBP/222 + MiBP/222. BMI, body mass index. CI, confidence interval; MEHP (mono-2-ethylhexyl phthalate); MEHHP (mono-(2-ethyl-5-hydroxyhexyl) phthalate); mono-(2-ethyl- 5-oxohexyl) phthalate (MEOHP); mono-(2-ethyl-5-carboxypentyl) phthalate (MECPP); mono-(3-carboxypropyl) phthalate (MCPP); monobenzyl phthalate (MBzP); monoethyl phthalate (MEP); monobutyl phthalate (MBP); and monoisobutyl phthalate (MiBP); RR, risk ratio. ^#^ *p* ≤ 0.10 and * *p* < 0.05.

**Table 3:** Associations between urinary phthalate biomarker concentrations and timing of uterine fibroid diagnosis.

	Fibroid Diagnosis ≥ 5 Years before Midlife Urine Collection (*n* = 111)	Fibroid Diagnosis < 5 Years before Midlife Urine Collection (*n* = 82)
Phthalate Biomarker	OR (95% CI)	OR (95% CI)
∑DEHP ^1^	1.18 (0.95, 1.45)	1.29 (1.03, 1.61) *
MCPP	0.96 (0.83, 1.11)	1.01 (0.86, 1.18)
MBzP	1.06 (0.87, 1.28)	0.84 (0.68, 1.05)
∑Plastics ^2^	1.17 (0.93, 1.46)	1.23 (0.96, 1.56) ^#^
MEP	0.98 (0.86, 1.11)	1.08 (0.93, 1.26)
MBP	1.02 (0.80, 1.31)	1.16 (0.91, 1.49)
MiBP	1.14 (0.91, 1.44)	1.16 (0.91, 1.49)
∑PCP ^3^	1.00 (0.84, 1.20)	1.19 (0.99, 1.43) ^#^
∑Phthalates ^4^	1.06 (0.86, 1.33)	1.30 (1.05, 1.61) *
∑AA ^5^	1.20 (0.93, 1.55)	1.35 (1.04, 1.76) *

Multinomial logistic regression models evaluated the odds of being diagnosed with fibroids ≥5 years (*n* = 111) or <5 years (*n* = 82) before baseline compared to never being diagnosed with fibroids (*n* = 519) for every 2-fold increase in phthalate biomarker concentration. Models account for race/ethnicity, income, age at menarche, oral contraceptive use, parity, fertility consultation, and midlife body mass index. ^1^ ∑DEHP = MEHP/278 + MEHHP/294 + MEOHP/292 + MECPP/308; ^2^ ∑Plastics = MEHP/278 + MEHHP/294 + MEOHP/292 + MECPP/308 + MCPP/252 + MBzP/256; ^3^ ∑PCP = MEP/194 + MBP/222 + MiBP/222; ^4^ ∑Phthalates = MEHP/278 + MEHHP/294 + MEOHP/292 + MECPP/308 + MCPP/252 + MBzP/256 + MEP/194 + MBP/222 + MiBP/222; ^5^ ∑AA = MEHP/222 + MEHHP/294 + MEOHP/292 + MECPP/308 + MBzP/256 + MBP/222 + MiBP/222. CI, confidence interval; OR, odds ratio. ^#^ *p* ≤ 0.10 and * *p* < 0.05.

## Data Availability

The data presented in this study are available on request from the corresponding author. The data are not publicly available because participants did not consent to share data on public websites.
